# Sex differences in resting hemodynamics and arterial stiffness following 4 weeks of resistance versus aerobic exercise training in individuals with pre-hypertension to stage 1 hypertension

**DOI:** 10.1186/2042-6410-2-9

**Published:** 2011-08-25

**Authors:** Scott R Collier, Vincent Frechette, Kathryn Sandberg, Patrick Schafer, Hong Ji, Harold Smulyan, Bo Fernhall

**Affiliations:** 1 Vascular Biology and Autonomic Studies Laboratory, Appalachian State University, 111 Rivers Street, Boone, NC 28608, USA; 2 Department of Medicine, SUNY Upstate Medical University 750 East Adams Street, Syracuse, NY 13210, USA; 3 Department of Kinesiology and Community Health, University of Illinois at Urbana-Champaign, 906 South Goodwin Ave, Urbana IL 61801, USA; 4 Center for the Study of Sex Differences in Health, Aging and Disease, Georgetown University, 4000 Reservoir Road, NW, Washington, DC 20057, USA

## Abstract

**Background:**

Hypertension (HTN) exhibits sexual dimorphism; the incidence for women surpasses men during the sixth decade of life, while the pharmacological treatments are less effective and produce more side-effects in women than in men. Aerobic exercise (AE) has been shown to prevent and treat HTN; however, resistance exercise (RE) is not recommended as a strategy to treat HTN. In this study, we investigated the potential sex differences of AE versus RE in a cohort of unmedicated patients with hypertension.

**Methods:**

In total, 40 moderately active, pre-hypertensive or stage 1 essential hypertensive male (M) and female (F) participants aged 40 to 60 years were randomly divided into four groups: M AE, M RE, F AE, and F. Each group exercised at moderate intensity, 3 days/week for 4 weeks. Hemodynamic, vascular and blood-flow data were collected before and after exercise training.

**Results:**

Men showed a significant increase in central pulse wave velocity following RE while females showed no significant changes (12 ± to 13.9 ± vs. 9.2 ± to 9.6 ± m/s, respectively). RE showed significantly greater increases in peak blood flow when compared to AE (F RE 15 ± to 20 ± vs. F AE 17.5 ± to19.5 ±, M RE 19 ± to 24 ± vs M AE 21 ± to 25 ± ml* 100 ml*min, respectively). In addition, systolic and diastolic BP decreased greater for women following RE when compared to AE whereas men showed comparable decreases in BP following either exercise mode.

**Conclusion:**

Moderate-intensity RE training may be a more favorable for women as a treatment option for hypertension because of greater decreases in diastolic BP and significant increases in flow-mediated dilation without concomitant increases in arterial stiffness, compared with their male counterparts.

## Background

Hypertension exhibits sexual dimorphism; women have lower systolic blood pressure (SBP) levels than men from puberty through their mid-fifties, whereas the opposite is true after the sixth decade of life [1]. Hypertension is not only more prevalent in women than in men, but also more severe and less amenable to control, especially in older women [2].

Moderate-intensity aerobic exercise (AE) is recommended as a non-pharmacological therapy for the management of raised BP because it reduces both SBP and diastolic blood pressure (DBP), and improves arterial function in pre- to stage 1 essential hypertension in both sexes [3]. Our laboratory previously showed that resistance exercise (RE) also reduced BP but to a slightly lesser extent than AE in a study with a predominantly male population [3]. RE training may be of greater importance to women because this mode of training increases bone-mineral density, which has been shown to protect against osteoporosis [4,5], and serves to increase muscle strength and endurance, which can prevent or delay the risk of disability in aging women [5,6]. However, RE training may also increase arterial stiffness, which would be a deleterious effect. The only available study in unmedicated patients with hypertension showed that arterial stiffness increased after RE [3]; however, this study was conducted in a primarily male population.

Recently, it has been shown that older men have increased blood flow after AE training, and that endothelial function is preserved in trained subjects after acute RE, which may decrease resting BP even in the presence of increases in arterial stiffness [7,8]. This is an important point to consider for individuals presenting with hypertension because many have increased arterial stiffness resulting from their condition. Although many studies have been conducted in normotensive and exercise-trained populations, relatively little is known about the effect of RE training on BP and arterial function in women with hypertension. Therefore, the purpose of this study was to compare the effects of 4 weeks of AE versus RE in both women and men with hypertension who were not on anti-hypertensive medications. We hypothesize that women would show greater increases in blood flow and greater decreases in BP without concomitant increases in arterial stiffness.

## Methods

The protocol was approved by the University institutional review board, and all subjects gave written informed consent before participation.

### Subjects

In total, 40 moderately active subjects (20 men, 20 women) aged 40 to 60 years, who were pre-hypertensive (120 to 139 systolic and/or 80 to 89 mmHg diastolic BP) or or had stage 1 essential hypertension(140 to 149 systolic and/or 90 to 99 mmHg diastolic) were recruited by local physicians and screened for medical and health histories [9]. The female group consisted of postmenopausal women only, to avoid the influence of hormonal changes, and they were required to have a history of more than 12 months of amenorrhea and no use of hormone-replacement therapy. Subject baseline characteristics are shown in Table 1.

**Table 1:** Descriptive characteristics^a^

	RE M	AE M	RE F	AE F
Age, years	44 ± 1.5	46 ± 1.5	52 ± 1.5*	54 ± 1.5*
Stature cm	177 ± 1*	179 ± 1*	160 ± 1	162 ± 1
Mass, kg	101 ± 5*	97 ± 5*	72 ± 5	78 ± 5
Body fat, %	31 ± 2	29 ± 2	40 ± 2*	37 ± 2*
SBP, mmHg	138 ± 3.2	136 ± 4.2	148 ± 6*	147 ± 4.5*
DBP, mmHg	78 ± 2	80 ± 2	78 ± 3	81 ± 2
MAP, mmHg	96 ± 2	99 ± 2	101 ± 2	103 ± 2
RHR, beats/min				
Before exercise	71 ± 3	75 ± 3	67 ± 5	69 ± 4
After exercise	73 ± 3	68 ± 3	68 ± 5	66 ± 4

Abbreviations: AE, aerobic exercise; DBP, diastolic blood pressure; F, female; RE, resistance exercise, M, male; MAP, mean arterial pressure; RHR, resting heart rate; SBP systolic blood pressure.

All data are expressed as mean ± SE;

Asterisk (*) denotes significance (*P *≤ 0.05).

On average, men are diagnosed with hypertension at an earlier age than women, therefore age-matched men might have undergone structural and functional changes that would confound our results. Thus, we chose to consider how long a subject had lived with the disease. Each subject consented to their blood-pressure history being provided by their physician, including the length of time the subject had been at or above pre-hypertension levels. All subjects enrolled in the study had been recently diagnosed (within 1 year) as having pre-hypertension or stage 1 essential hypertension. Consequently, women were significantly older than men (8 years).

Using the medical histories obtained from physician records, it was confirmed that no subjects had a history of diabetes, kidney disease, atherosclerosis or hypercholesterolemia. None of the subjects was taking any medication, including antihypertensives or aspirin, and all were non-smokers.

### Study procedure

Subjects reported to the laboratory at the same time of day, 3 hours after a meal, for each of the three measurement visits. Subjects were asked to refrain from caffeine and exercise before each visit. The first visit consisted of group randomization, and the taking of a health history and physical activity questionnaire, followed by tests of body composition, peak aerobic capacity (VO_2peak_) or 10 repetition maximum (10 RM) test, and measurement familiarization.

During the second visit, after 15 minutes of supine rest in a dimly lit, quiet room, pulse wave velocity (PWV) and BP, followed by blood flow and reactive hyperemia were measured while the subject rested quietly. During the following 4 weeks, all subjects reported to a training facility, and each exercise session was supervised by an exercise physiologist. All subjects reported back to the laboratory for their post-training measurements at between 24 and 48 hours after completion of the last exercise session. All measurements were repeated at the same time of day in the postprandial state (>3 hours) and in the same order as previous measurements.

### Anthropometrics

Whole-body plethysmography (Bod Pod, Life Measurement Inc., Concord, CA, USA) was used to assess body composition [10], and body weight was measured using the Bod Pod scale to the nearest half-kilogram. Height was measured using a stadiometer to the nearest 0.5 cm, and body mass index was calculated as: weight (kg) divided by height (m^2^).

#### Maximal exercise testing

Subjects randomized to the AE arm of the study completed a customized VO_2peak _protocol on a treadmill. Briefly, subjects started walking at 3 miles/hour for 2 minutes. Adjustments to the speed were made until the subject achieved a stable pace, at which point the grade was increased by 2% every 3 minutes until volitional fatigue was reached. Expired gases were analyzed using a breath-by-breath metabolic cart (Quark b^2^; Cosmed, Rome, Italy). Rating of perceived exertion (RPE) and heart rate (HR) (Polar Electro Inc., Woodbury, NY, USA) were acquired once per stage. Maximal effort was achieved when subjects met three of the following criteria: 1) a final RPE score of ≥17 on the Borg scale (rankings from 6 to 20); 2) a respiratory equivalent ratio (RER) of >1.15; 3) no change in HR after a change in workload; and/or 4) a plateau in oxygen uptake with an increase in workload (<150 ml). The AE group underwent maximal VO_2peak _testing both before and after training.

Subjects randomized to the RE arm completed a 10 RM test. After a brief warm-up, an estimated load was given for each exercise (leg press, lateral pulldown, leg extension, chest press, leg curl, shoulder press, bicep curl, tricep press, and abdominal crunch), and each subject completed no fewer than 8 and no more than 15 repetitions. If the subject achieved fewer or more than 10 repetitions, prediction tables were used to add or reduce weight until the preferred load was attained. The subjects were again measured at the conclusion of their RE program to evaluate the effectiveness of the training protocol.

### Exercise training

The AE training consisted of 30 minutes of treadmill exercise at 65% of the subject's previously determined VO_2peak_, 3 days per week for 4 weeks. The 10 RM test provided the basis for individual load resistance for the dynamic RE sessions. The REs consisted of leg press, chest press, leg extension, lateral pulldown, leg curls, shoulder press, bicep curl, tricep press and abdominal crunch, all performed on the same equipment (Life Fitness, Schiller Park, IL, USA). Each subject completed three sets of ten repetitions at 65% of their 10 RM, 3 days/week for 4 weeks. Each RE session took approximately 45 to 50 minutes to complete. Subjects were asked to refrain from any exercise outside of their AE or RE sessions.

### Central and peripheral arterial stiffness

PWV measurements were obtained with two MD6 bidirectional transcutaneous Doppler probes (Hokanson, Bellevue, WA, USA) in accordance with the guidelines of the Clinical Application of Arterial Stiffness Task Force 3 [11]. Each subject was monitored by electrocardiography (modified CM5), and HR data were gated in phase with the PWV measurements and used as timing markers for PWV identification. Central PWV measurements were obtained from the left common carotid artery to the left femoral artery. Distances from the carotid site to the mid-point of the manubrium sterni were subtracted from the carotid-to-femoral artery distance. Peripheral PWV measurements were obtained from the left femoral artery to the ipsilateral superior dorsalis pedis artery. The distance between each PWV location was obtained with a tape measure, and recorded to the nearest millimeter.

Data were collected in real time by aligning the Doppler waveforms and the electrocardiography tracings on a computer screen (MP100, BioPac Systems, Santa Barbara, CA, USA). All data were stored until analyzed at a later time. PWV was measured from the foot-to-foot flow wave velocity, whereas the foot of the sound wave was identified as the point of systolic upstroke. A minimum of 10 pulse contours were recorded and analyzed as the distances between points, and the time delay between proximal and distal foot waveforms was calculated as the distance (D) divided by the change in time (m/s). In our laboratory, one blinded technician analyzed all of the data, and the intra-class correlation coefficient for PWV, calculated using both central and peripheral sites on two separate days, was 0.98.

### Hemodynamic monitoring

In addition to physician visits, BP was measured twice (before and after training) through standard manual methods after the subject had rested quietly for 15 minutes in the seated position. During supine rest, BP was taken before and after the intervention with an automated auscultatory instrument (SunTech Medical Instruments, Raleight, NC, USA) method. All BP measurements were in accordance with American Heart Association standards. HR was taken from the calculation of successive R-R intervals from the three-lead electrocardiograph.

### Blood flow and reactive hyperemia

Forearm blood flow (FBF) and forearm reactive hyperemia (FRH) were measured using a mercury-in-silastic strain-gauge plethysmograph (EC-6, DE Hokanson, Inc., Issaquah, WA, USA) as described previously [12]. All data were transmitted to a computer, and analyzed (Non-Invasive Vascular Program 3 Software Package, version 5.27b; DE Hokanson). Six plethysmography measurements were averaged for baseline blood-flow values before and after exercise. The intra-assay coefficient of variation was 5.2%. Following baseline flow measurements, a second occlusion cuff was placed over the first upper-arm cuff and inflated for 5 minutes at 250 mmHg. Again, 1 minute before deflation of the upper-arm cuff, a wrist cuff was inflated to 50 mmHg above SPB, which remained inflated until the end of the measurement. After 5 minutes of occlusion, the upper-arm cuff was rapidly deflated to induce reactive hyperemia. Following cuff release, 3 minutes of blood-flow measurements were recorded as described above. FBF was expressed as milliliter/min/100 ml of tissue. To avoid confounding other vascular measurements, RH was conducted after all other measurements were complete.

### Treatment of the data

Vasodilatory capacity was calculated from the blood-flow data as the area under the curve (AUC) above baseline values using GraphPad Prism software (version 3.02;) and the trapezoidal rule on the basis of actual datum points. A one-way analysis of variance (ANOVA) with repeated measures (exercise mode (AE versus RE) by time (before versus after training) by sex (male versus female)) was used with SPSS software (version 17; SPSS Inc. Chicago, IL, USA) on all dependent variables. If a significant interaction was detected, an appropriate *post hoc *test was conducted. The *a priori *significance was set at α < 0.05, and all data are reported as means ± SEM.

The sample size of 40 subjects for the present study was based on previous data from our laboratory gathered under similar conditions. For these calculations, the STATA statistical software package (STATA, College Station, TX, USA) was used to determine the number of subjects to give us adequate statistical power at *P *< 0.05.

## Results

Women had a significantly greater percentage of body fat and higher SBP than their male counterparts, while men had significantly greater stature and mass (RE 29 kg, AE 19 kg) than women.

**Figure 1:**
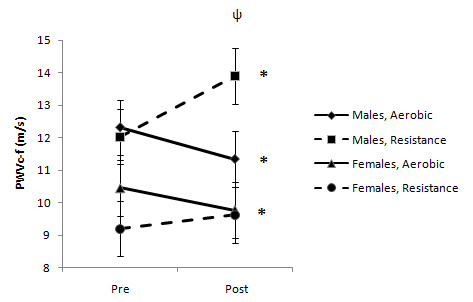
**Changes in central pulse wave velocity (PWV) between men and women**. There was a significant time × sex by mode interaction for central PWV (ψ, *P *= 0.001). Men had a significant increase in central PWV after resistance exercise (RE), whereas PWV in women did not change over time. Further, there was a significant decrease in PWV in both aerobic exercise (AE) groups (*P *= 0.03).

There was a significant time × sex × mode interaction for central PWV *P *= 0.001). Men had a significant increase in central PWV after RE, whereas women had no change in central PWV over time (Figure 1). Further, there was a significant decrease in PWV in both AE groups (*P *= 0.03). There were no significant sex differences in peripheral PWV (M AE 12.8 ± 1.9 versus 12.7 ± 1.9 RE 13.2 ± 1.8 versus 13.9 ± 1.8, F AE 10.3 ± 1.6 versus 10.3 ± 1.6 and F RE 9.4 ± 1.5 versus 9.6 ± 1.5 m/s, before and after training respectively) after RE or AE training.

**Figure 2:**
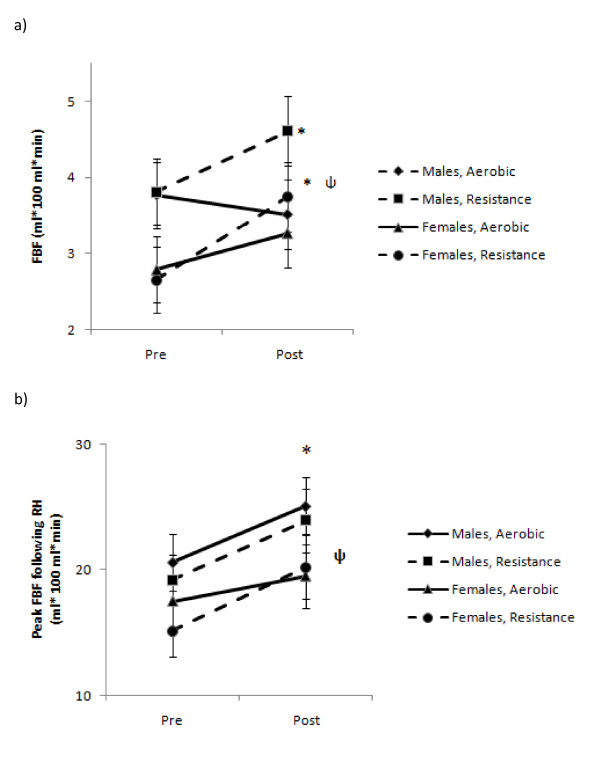
**Changes in basal and peak forearm blood flow (FBF)**. **(A) **For basal FBF, there was a significant time × mode × sex interaction (ψ, *P *= 0.01) as resistance exercise (RE) produced greater increases in basal FBF compares with aerobic exercise (AE), and women had a significantly greater increase after RE than men (*P *= 0.042). **(B) **For peak FBF, there was a significant increase in peak blood flow after both exercise modes, and RE induced significantly higher peak blood flow (*P *= 0.04).

There was a significant time× mode × sex interaction (ψ, *P *= 0.01) as there was a greater increase in FBF after RE than after AE, and women had a significantly greater increase than men after RE (*P *= 0.04 Figure 2a). There was a significant increase in peak blood flow after both modes of exercise, but RE condition resulted in significantly higher peak blood flow (*P *= 0.04; Figure 2b). There was a significant increase in AUC of blood flow after training, and RE increased blood flow significantly more than AE in both cohorts (M AE 94.8 ± 10 versus 128.7 ± 12, M RE 89.2 versus 139.4 ± 12, F AE 78.3 versus 97.3 ± 9 and F RE 79.4 ± 10 versus 110.6 ± 12 ml/100 ml^/^min, before versus after training, respectively).

**Figure 3:**
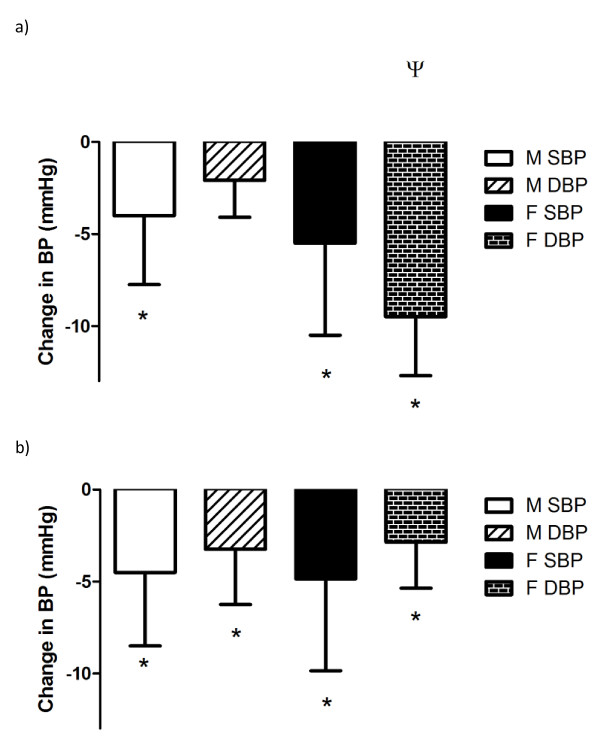
**Changes in blood pressure (BP) after exercise**. **(A) **Resistance exercise (RE) induced significant decreases in resting systolic blood pressure (SBP) (*P *= 0.045) but not diastolic blood pressure (DBP) for men, and a group × mode × time interaction for DBP for women (ψ, *P *= 0.02). **(B) **AE showed significant training effects in both cohorts (*P *= 0.048) yet no significant group × mode differences nor a significant decrease in [DBP].

RE induced significant decreases in resting SBP (*P *= 0.045) but not DBP for men, and there was a group × mode × time interaction for DBP in women (ψ, *P *= 0.02; Figure 3a). Whereas AE produced significant training effects in both cohorts (*P *= 0.048), there were no significant group × mode differences or any significant decrease in DBP after RE training (Figure 3b).

## Discussion

The results from this study suggest that AE has different effects on the cardiovascular system of men and women. The majority of studies assessing the effects of exercise training on BP and arterial function have been conducted in single-sex or mixed-sex populations, but without direct comparisons between the sexes [13-17]. To our knowledge, the present study is the first to directly compare differences between the sexes in the effects of exercise mode on resting hemodynamic parameters and variables of arterial stiffness in an unmedicated hypertensive cohort. The major novel finding of our study was that RE resulted in increases in central arterial stiffness in men, but not in women (Figure 1a,b). Conversely, AE produced a decrease in central arterial stiffness but no change in peripheral arterial stiffness for either sex. Another novel finding was that compared with AE, RE resulted in a greater increase in basal and peak FBF in both men and women (Figure 2a,b).

Several studies support our present findings on arterial stiffness. Miyachi *et al. *[18] reported a 19% increase in central arterial stiffness in a group of men who underwent a 4-month RE protocol [18]. Similarly, Bertovic *et al. *[19] found that men who used RE training regularly (consistently for a period of >12 months) had stiffer arteries than did their sedentary counterparts. One researcher reported RE with the upper body has been shown to increase arterial stiffness in pre-menopausal women [20], however, multiple investigations have shown RE to elicit no change in arterial stiffness in middle-aged women [21,22]. Not all studies have shown RE to yield increases in arterial stiffness in men and no change or a decrease in stiffness in women [21,23,24]. Cortez-Cooper *et al. *[25] found that high-intensity RE elicited an increase in arterial stiffness in women, whereas Rakobowchuk *et al. *[24] observed no change in arterial stiffness with RE in healthy men. However, none of these previous studies evaluated the effect of RE in an untreated hypertensive cohort.

Conversely, AE elicits favorable effects on the vasculature in both men and women. Moderate-intensity AE has been shown to increase arterial stiffness in older men [26] and in postmenopausal women [27]. Arterial stiffness increases with aging in sedentary individuals, yet this increase is attenuated with regular AE [28]. However, there is some evidence of a threshold effect, at which exercise becomes less effective in combating age-related increases in arterial stiffness. Ferrier and colleagues [29] performed a study examining the effects of moderate-intensity exercise on arterial stiffness in an older mixed-sex population (mean age 64.7 years) with isolated systolic hypertension, and found that 8 weeks of moderate-intensity cycling did not stimulate the positive changes in arterial stiffness seen with exercise in any of the aforementioned studies. This suggests that the deleterious changes occurring in the vasculature with aging may become irreversible if proper interventions are not introduced and such processes are allowed to progress over time. Interestingly, women seem to have greater age-related increases in ventricular/vascular stiffness than men, even in the absence of cardiovascular disease [30]. This age-dependent increase in arterial stiffness with aging in women is believed to be a contributor to the accelerated increase in SBP that is prevalent in older women as they age. Thus, it is important to understand the effect of potential non-pharmacological therapies, such as exercise training, on both BP and arterial stiffness. It is important to note that many investigators have shown increases in central arterial stiffness after RE, but no changes in peripheral stiffness. This is important because the arterial baroreflex found in the aortic arch may be susceptible to desensitization if central arterial stiffness increases. This would lead to chronically higher sustained pressure and deleterious effects on the systemic arterial circulation. Importantly, despite discrepancies between men and women in exercise-induced arterial stiffness, both modes of exercise we used elicited beneficial changes in BP, some of which were similar to those seen in our previous study [3]. AE elicited a decrease of 4 mmHg SBP and 3 mmHg DBP in men and decreases of 5 and 3 mmHg in SBP and DBP, respectively in women (Figure 3a,b). These changes mirror those seen in a meta-analysis by Fagard on the effects of AE on BP [31]; RE yielded decreases in SBP and DBP of 4 and 2 mmHg, respectively. These changes were similar to those seen in the meta-analysis by Kelley and Kelley [32]. Women also exhibited decreases in SBP and DBP with RE, but had a much greater decrease in DBP compared with men: -10 versus -2 mmHg. This could be explained by the difference between the sexes in arterial stiffness after RE, as women did not exhibit an increase in arterial stiffness with RE. In addition, the lower muscle mass in women may derive less retrograde shear stress from RE compared with men, which may augment greater functional changes because of the attenuation of the blunted signaling on the endothelium [33,34].

The decrease in mean arterial pressure seen in men despite the increase in arterial stiffness suggests that the increase in flow, potentially caused by augmented vasodilation, is a protective compensatory mechanism. However, the flow increases with RE were identical in men and women. Furthermore, in both sexes, RE induced a greater increase than AE in basal FBF. It is currently known that functional changes resulting in increases in vascular conductance are endothelium-dependent, and are modulated by shear stresses on the vascular wall, augmenting the expression of endothelial nitric oxide synthase and extracellular superoxide dismutase, which act synergistically to increase the bioavailability of nitric oxide (NO) [35-37]. It is possible that the high force contractions produced by the skeletal muscle in RE may yield greater shear stresses on the vasculature, resulting in an increase in NO bioavailability at rest. Interestingly, the changes in basal FBF with RE did not translate to a concomitant increase in peak vasodilatory capacity in men, as peak FBF after RH was nearly identical with both modes of exercise. However, the changes in female RH were greater after RE than AE, which may result in greater blood flow and greater decreases in BP compared with their male counterparts.

## Conclusion

This investigation shows that both AE and RE elicit beneficial changes in resting BP in both men and women. However, RE produced increases in central arterial stiffness in men with hypertension, whereas no such changes were shown in the women. These changes in stiffness were offset by a concurrent enhancement in vascular conductance, as RE elicited greater increases in blood flow compared with AE in both sexes. Therefore, RE can be a beneficial addition to the treatment plan in both men and women with hypertension. With exercise being a beneficial non-pharmacological intervention in the treatment of hypertension, we believe it to be important to uncover any sex differences in the effects of exercise, as this could provide valuable information allowing practitioners to develop more efficient treatment plans for both men and women with hypertension.

## Competing interests

The authors declare that they have no competing interests.

## Authors' contributions

SC conceived of the study, collected data and wrote the manuscript; VF recruited subjects, aided in study conception and provided clinical insight for the paper; KS helped in the study conception, ran all of the assays, edited the paper and provided clinical knowledge; PS helped in data collection and writing of the manuscript; HJ ran all of the assays and helped with the Methods section of the paper; HS aided in study conception and provided clinical insight for the paper; and BF helped with the study conception and manuscript editing. All authors read and approved the final manuscript.
